# 101st EGPRN meeting, 16–19 October 2025, Plovdiv-Bulgaria: Empowering the next generation of family physicians in a changing healthcare landscape

**DOI:** 10.1080/13814788.2026.2625569

**Published:** 2026-03-27

*Every generation imagines itself to be more intelligent than the one that went before it, and wiser than the one that comes after it.*

*George Orwell*

The theme of this meeting, **‘Empowering the next generation of family physicians in a changing healthcare landscape’,** invites us to reflect on how we equip, support and inspire early-career general practitioners to become active contributors to research and innovation. As health systems across Europe evolve, family medicine stands at a crossroads – facing new demands, technological transformation and the imperative to deliver care that is both person-centred and responsive to community needs.

This evolving landscape brings new opportunities. It calls for more **inter-professional collaboration**, with a focus on how to structure it effectively, and for deeper engagement with **multicultural realities**, including research into ethnic diversity, differential responses to treatment and culturally sensitive care approaches.

This meeting highlights the complexities and potential of research focused on empowering the next generation of family physicians. General practitioners work in close partnership with patients, colleagues and communities, and are uniquely positioned to generate practice-based research that reflects the realities of daily care. Navigating uncertainty, embracing diversity and responding to systemic changes are central to our discipline – and also fertile ground for inquiry.

By sharing evidence and lived experience, we aim to strengthen the research foundations of family medicine and inspire new perspectives for a changing world.

