# Supplementary material for: 101st EGPRN meeting, 16–19 October 2025, Plovdiv-Bulgaria: Empowering the next generation of family physicians in a changing healthcare landscape
Source: Eur J Gen Pract. 2026 Mar 27;32(1):2625569. doi: 10.1080/13814788.2026.2625569 (PMC13034704; doi:10.1080/13814788.2026.2625569)
Supplement: Related texts

# Abstract: Primary care at a crossroads: Advancing research on inter-professional collaboration and on integrated, equitable and complex care

**Authors:** Paul van Royen

**Affiliations:** Department of Family Medicine and Population Health, Faculty of Medicine and Health Sciences, University of Antwerp, Belgium

## Abstract

Primary care is undergoing a fundamental transformation, driven by increasing care complexity and technological innovation, persistent health inequities, the need for integrated, inter-professional models and more cultural competence. This keynote explores with current projects how future-ready primary care research must evolve to support and guide this shift. It advocates for more novel research methodologies, including longitudinal mixed-method studies, implementation science, complexity science, pragmatic trials, realist evaluation and network analysis. These approaches are essential to better understand and support task shifting, mental–physical health integration and equitable care for vulnerable and multicultural populations in a changing healthcare landscape.

# Abstract: Empowering the next generation of family physicians in an everchanging healthcare landscape in Bulgaria. A take on the topic

**Authors:** Gergana Foreva

**Affiliations:** Department of General Practice, Medical University Plovdiv, Plovdiv, Bulgaria

## Abstract

We are at the end of the third decade of the reform of the healthcare system in our country, which introduced general medical practice, performed by a general medicine specialist. Despite the difficulties in transforming the healthcare system, general practitioners found their place. And if in the first decades the focus was entirely on changes and transition, structuring and the relationship with the national health insurance fund, gradually space opened up for new priorities.

In our country, the academic representation of the specialty took place in conjunction with the change in the health sector. This allowed scientific research to be done at the same time, reflecting the development of the specialty and its practical dimensions. When reviewing the dissertations and publications on general medicine in the Bulgarian scientific medical periodicals, the topics cover a wide spectrum – health service, clinical issues, education, etc. Since the establishment of the departments, the members have been regular participants in the EGPRN meetings. A major step was the accreditation of training practices in which general medicine residents work on an employment contract. With the newly adopted programme, research training became part of the residents’ curriculum.

Every generation has its own view of the world. And the landscape of the healthcare system has changed radically in recent decades. We work within the framework of a developing national health information system. It includes patients’ data concerning their health status. On the other hand, artificial intelligence is both at our and at the patients’ disposal if a question arises, and even more so in the hands of a generation native to technology. New realities facilitate and complicate daily work. Doctor–patient communication has always been an essential defining feature of the specialty, and the advent of new technologies is significantly changing its perspective. Generational diversity has its advantages, because different perspectives can be an important strength in defining our new health care system.

# Abstract: Artificial intelligence and health equity in primary care: A scoping review

**Authors:** Petroula Delliou, Elias Kondilis, Elias Sakellariou, Magda Gavana

**Affiliations:** Medical School, Aristotle University of Thessaloniki, Thessaloniki, Greece

**Keywords:** Primary health care, artificial intelligence, health equity, health services

## Abstract

**Background:**

Integrated primary healthcare (PHC) ensures that all individuals have access to health services. Artificial Intelligence (AI) has significantly transformed PHC by enhancing the quality, efficiency and reach of care. However, concerns have emerged regarding AI’s potential to either reduce or exacerbate health inequities, one of the most persistent challenges in healthcare systems globally. Understanding AI’s role is essential for equipping the next generation of family physicians with the knowledge and insights needed to lead equitable and innovative care.

**Research questions:**

The primary objectives of the research revolve around two main questions: (a) how AI affects health equity in the PHC setting and (b) what the contribution of AI in PHC to health inequalities is.

**Method:**

A scoping review was conducted with literature research across PubMed, Scopus, IEEE Xplore databases and grey literature sources such as JSTOR and Google Scholar, covering the period from 2000 to 2025. Article selection adhered to the PRISMA-ScR guidelines, and thematic analysis was used to synthesise findings.

**Results:**

Out of 1,211 identified publications, 25 met the inclusion criteria. The results were categorised into 11 thematic domains, reflecting both positive and negative impacts of AI on health equity: (1) improving access to healthcare and addressing the digital divide, (2) enhancing early disease detection in underserved populations, (3) reducing disparities in clinical decision-making, (4) agency for self-care, (5) algorithmic bias, (6) ethical concerns, (7) patient trust, (8) dehumanisation and biomedicalisation, (9) patient–doctor relationship, (10) participatory approaches and community involvement and (11) provider acceptance, opportunity loss and equity.

**Conclusions:**

This review summarises the extent to which implementation of AI in PHC promotes health equity or mitigates health inequalities and highlights the urgent need for further research to ensure its equitable implementation in healthcare systems and better prepare and empower future family physicians to navigate and lead in a rapidly transforming healthcare environment.

# Abstract: Fostering scholarship in GP training in Ireland; a national initiative to build and enhance research engagement

**Authors:** Aileen Barrett, Darach Brennan, Stephanie Dowling, Stephen Brennan, Ciaran Foley, Ruth Barrett

**Affiliations:** Irish College of GPs, Dublin, Ireland

**Keywords:** GP training, scholarship, academic careers, quality improvement

## Abstract

**Background:**

Scholarship, encompassing clinical audit, quality improvement and research, is a cornerstone of general practitioner training, yet trainee engagement remains limited. Our recent work has indicated the need for additional career opportunities and skills to support varied and enhanced careers, including those related to academic general practice.

**Research questions:**

What range of trainee and supervisor resources and organisational support are required to build research capacity on a national scale in general practice training in Ireland?

**Method:**

The project adopted a quality improvement approach, engaging stakeholders through interviews, presenting findings and generating faculty development supports in the first PDSA cycle. The second cycle generated national standards for the conduct and reporting of trainee clinical audits, QI projects and research studies, emphasising structured methodologies and ethical rigour. This cycle culminated in recommendations for ‘structures’ (e.g. protected time, regional research days), ‘processes’ (e.g. standardised modules, proposal timelines) and ‘resources’ on a national level.

**Results:**

Supervisor frustration with research ethics processes dominated supervisors’ perceptions of the challenges around trainee engagement in research; this outcome resulted in a new webinar for trainees and discussions with the REC around approval for non-interventional studies. In PDSA II, all proposed project standards achieved consensus (>60%) for inclusion and included the use of clearly defined features for each project type (e.g. complete audit cycles, SMART objectives for research). Engagement in faculty development initiatives, designed in Phase I, was limited, prompting a shift to e-learning. Trainee resources, such as blended learning modules and REC application webinars, were implemented.

**Conclusions:**

This project has provided a structured blueprint to address barriers such as supervisor confidence and time constraints. The resources needed include expanding e-learning, fostering cross-scheme collaboration and supervisor skills development.

# Abstract: Inter-professional learning from the experience of patient-mentors

**Authors:** Alain Mercier, Olivia Gross, Yannick Ruelle, Rémi Gagnayre

**Affiliations:** Université Sorbonne Paris Nord, Bobigny, France

**Keywords:** Interprofessional education, epistemic injustices, incorporated knowledge, transformational learning

## Abstract

**Background:**

Informal and hidden curricula as well as the positivist paradigm partly explain the decline in empathy observed among health students. They also generate epistemic inequities that are exerted on patients but also between health professionals. An inter-professional programme involving patient-mentors was set up as part of the initial training of health students to improve this situation.

**Research questions:**

How does the programme contribute to achieving its intended objective, and what are the underlying mechanisms that help identify its active components?

**Method:**

Forty-five health students from six different training courses were brought together in small groups on four occasions with a patient-mentor. The qualitative study combined individual and group interviews as well as written responses to a questionnaire.

**Results:**

These meetings acted as a trigger for transformational learning. The students became aware of some of their prejudices and limitations, which should foster more horizontal relationships with patients and other health professionals. The self-management of the groups, the fact that the number of participants in these groups was relatively small, the multiple encounters with a patient outside care and the suggested themes for discussion enabled the updating of learning mechanisms. The latter refer to decentration, humility, non-judgement and closeness, which were actualised in these groups. The development of autonomous thinking and the consideration of their own feelings can be seen as intermediate effects. The final effects are the development of embodied knowledge of the patient-centred approach and an increased sense of responsibility. Both dimensions are manifested in new resolutions and new professional practices.

**Conclusions:**

The study explains the learning processes of the students in teaching of this order, but it also shows that transformational learning is still possible at this stage of their studies.

# Abstract: ‘It’s all a struggle and an effort’ – Experiences of people with post-COVID-syndrome dealing with their symptoms

**Authors:** Uta Sekanina, Imke Koch, Franziska Grimm, Nils Schneider, Frank Mueller

**Affiliations:** University Medical Center Goettingen, Göttingen, Germany

**Keywords:** Post-COVID syndrome, general practitioner, coping strategies, healthcare system experiences, qualitative research

## Abstract

**Background:**

Following a COVID-19 infection, some people develop long-term symptoms such as fatigue or shortness of breath – symptoms of post-COVID Syndrome (PCS). These PCS patients face considerable challenges in everyday life, at work and within the healthcare system. General practitioners (GPs) play a pivotal role in providing care to PCS patients, as they are often the first point of contact.

**Research questions:**

This study aims to examine how patients with PCS cope in everyday life and their experiences within the healthcare system, with a particular focus on the care provided by GPs.

**Method:**

This qualitative study is part of a larger mixed-methods research project on PCS. Participants were recruited via a random sample of people diagnosed with PCS and insured by a German sickness fund. The research team conducted 26 semi-structured interviews. The audio-taped and transcribed interviews were analysed using qualitative content analysis according to Kuckartz and Rädiker (2022) in MAXQDA 2024.

**Results:**

PCS patients reported that they felt severely impaired by their symptoms in everyday life. Many were unable to work. Their mental health was negatively affected. They developed individual and social coping strategies, e.g. searching for information on PCS or re-distributing family tasks. Within the healthcare system, PCS patients often felt ignored or even stigmatised due to a lack of understanding and recognition of their persistent symptoms. However, some healthcare professionals, particularly GPs, displayed empathy and offered active support. This validation was highly beneficial for PCS patients’ illness adjustment.

**Conclusions:**

PCS patients struggle to manage their condition in everyday life and perceive support from the healthcare system as insufficient. GPs can lighten the observed emotional burden by taking PCS patients’ symptoms seriously and working with a symptom-oriented, biopsychosocial treatment concept. The study demonstrates methodological robustness due to random sampling, an appropriate sample size for qualitative research and systematic qualitative analysis.

# Abstract: Cis-heteronormativity and discrimination in primary care: A qualitative study on LGBTQI + healthcare

**Authors:** Stavroula Kostaki, Efthymia Makridou, Panagiota Karagianni, Ioanna Ierodiakonou-Benou

**Affiliations:** Aristotle University of Thessaloniki, Thessaloniki, Greece

**Keywords:** LGBTQI + health, cis-heteronormativity, inclusive primary healthcare

## Abstract

**Background:**

LGBTQI + individuals face well-documented disparities in access to healthcare, contributing to increased vulnerability to a range of diseases. Primary care, as the first point of contact for most patients, has the potential to either mitigate or reinforce these disparities. This study investigates how cis-heteronormative assumptions, that patients are cisgender and heterosexual, embedded in biomedical discourse and clinical practice result in discrimination against LGBTQI + individuals.

**Research questions:**

How does cis-heteronormativity manifest in healthcare practices, and how does it affect LGBTQI + patients’ access to and quality of primary care?

**Method:**

A qualitative study was conducted using 42 semi-structured interviews across three groups (*n* = 14 each): LGBTQI + patients, LGBTQI + healthcare professionals and non-LGBTQI + providers. Supplementary field observations were carried out in three outpatient clinics of public hospitals in Thessaloniki. Data were analysed using critical discourse analysis, informed by queer theory and the social determinants of health framework.

**Results:**

Non-cisgender and non-heterosexual identities are often pathologised or rendered invisible in the discourse and practices of psychiatry, gynaecology, endocrinology and infectiology. Lesbian and gay patients frequently receive reproduction-focused sexual health advice irrelevant to their needs. Trans individuals often avoid Pap tests due to non-inclusive procedures and must undergo psychiatric evaluation to access hormone therapy, reinforcing binary gender norms. LGBTQI + individuals may be referred to exploratory or corrective mental health interventions. Such practices either pathologise or dismiss LGBTQI + health needs in core areas of primary care, such as sexual and reproductive health, mental health and hormone-related treatment (initiation, follow-up or referral).

**Conclusions:**

Cis-heteronormativity acts as a structural barrier to equitable care within healthcare settings, reinforcing discrimination against LGBTQI + patients. Inclusive sexual histories, gender-neutral forms, organ-based screening, accessible gender-affirming care and welcoming environments are essential changes. With its holistic and patient-centred approach, primary care can play a critical role in promoting inclusivity and reducing health disparities.

# Abstract: Assessing family medicine residency training: Evaluation of resident perspectives

**Authors:** Ana Maria Alexandra Stanescu, Ioana Veronica Grajdeanu

**Affiliations:** “Carol Davila” University of Medicine and Pharmacy, Bucharest, Romania, Bucharest, Romania

**Keywords:** Residency, training, family medicine

## Abstract

**Background:**

As the demand for family physicians rises globally, ensuring that these doctors receive comprehensive, high-quality professional training becomes critical. The residency period is particularly important for shaping competent family physicians.

**Research questions:**

What are the current strengths, challenges and areas for improvement within family medicine residency training programmes from the perspective of medical residents?

**Method:**

We conducted a nationwide multicentric survey involving 12 university centres in Romania. The questionnaire, consisting of 55 items, explored residents’ satisfaction with their current training, identified areas needing improvement and assessed their preparedness and needs for future practice.

**Results:**

A total of 332 family medicine residents participated. Among them, 95.2% expressed intentions to practise in their home country, with 66% preferring urban areas. Working conditions influenced the specialty choice significantly. Satisfaction rates varied between 45.8% and 75.9% depending on the medical area. Notably, 87% cited complex legislation and authority-imposed restrictions as significant barriers. The majority indicated a desire for an extended cardiology rotation. Furthermore, 84% preferred hands-on training, whereas only 18.1% were engaged in research activities. Finally, 66.6% expressed interest in participating in international exchange programmes.

**Conclusions:**

We identified both strengths and weaknesses within family medicine residency training. Curricular adjustments and additional extracurricular opportunities are recommended to enhance the quality and effectiveness of residency training.

# Abstract: Assessing AI-generated smoking cessation advice for patient education in primary care

**Authors:** Canan Tuz, Bahar Ürün Ünal, Fethi Sada Zekey, Kübra Uyar Zekey, Demet Merder Coşkun, Tuncay Müge Alvur

**Affiliations:** Kocaeli University, Türkiye

**Keywords:** Smoking cessation, patient education, primary care, digital health

## Abstract

**Background:**

Artificial intelligence (AI) has emerged as a promising tool to support smoking cessation in primary care, particularly for populations underserved by traditional interventions. However, the quality of AI-generated smoking cessation advice remains understudied, especially in low-resource settings and among vulnerable groups such as adolescents.

**Research questions:**

This study aims to evaluate AI-generated responses to smoking cessation questions for patient education in primary care, comparing different AI programs in terms of knowledge, readability and quality.

**Method:**

Ten publicly accessible AI programs were prompted in Turkish with 24 standardised, open-ended smoking cessation questions framed as a patient consultation. Two family medicine specialists independently assessed each response’s readability using Ateşman’s Readability Index, reliability using the DISCERN instrument, and accuracy and motivational interviewing quality using a bespoke rubric and OARS (Open questions, Affirmations, Reflections, Summaries) framework. Inter-rater agreement was evaluated via intraclass correlation. Descriptive statistics were computed for readability scores, DISCERN ratings and accuracy grades.

**Results:**

All AI programs provided at least partially correct answers to all questions. The average readability score was 54.90 (medium difficulty) according to Ateşman’s Index. The mean DISCERN score was 66 ± 5.2, indicating excellent quality. Three AI programs incorporated core motivational interviewing skills. The most accurately answered question concerned e-cigarettes’ harm compared with traditional cigarettes, while medication advice was least evidence-based.

**Conclusions:**

Free AI chatbots deliver reliably accurate and moderately readable smoking cessation advice, supporting their potential role as patient education adjuncts in primary care—particularly for individuals with at least a high school education. Further research should compare AI-assisted versus clinician-led interventions on smoking-cessation outcomes.

# Abstract: Morbidity and survival profile of adults diagnosed with ASD compared with the general population – matched cohort study

**Authors:** Itamar Getzler, Ateret Malaachi, Orit Stoler

**Affiliations:** Maccabi Health Services, Haifa, Israel

**Keywords:** ASD, autism, morbidity, epidemiology

## Abstract

**Background:**

Autism spectrum disorder (ASD) is an early-onset and lifelong neurodevelopmental condition requiring continuous resources and support throughout life. Although there is substantial research concerning paediatric populations with ASD, the adult population remains underexplored.

**Research questions:**

What are the differences in morbidity characteristics between adults with ASD compared with the general population, and how can these inform preventive care strategies?

**Method:**

This is a retrospective cohort study, with a timeframe encompassing up to 26 years of follow-up, and included adult patients (>18 years) with an ASD diagnosis. A random control group was matched in a 3:1 ratio to the ASD group based on age, sex and socioeconomic status. Patient records were scanned to identify diagnoses of multiple diseases and their dates of diagnosis. Statistical analysis was performed to compare incidence and timing of diagnosis between the groups, and interaction between the variables.

**Results:**

The study cohort comprised 5326 men and 1639 women diagnosed with ASD with a mean age of 28 years. The hazard of developing diabetes mellitus type 2 (DM2), high blood pressure and inflammatory bowel disease diagnosis increased by 47%, 24% and 60%, respectively, by belonging to the ASD group versus the control group and with a lower age of diagnosis. The likelihood of being overweight was 49% higher in the ASD group than in the control group at any point during the study’s follow-up period and was correlated with psychiatric diagnosis. The risk of schizophrenia increased twofold. Incidences of COPD, cardiovascular diseases and bipolar disorder were not significantly different between groups.

**Conclusions:**

Our study highlights the distinct characteristics of adults with ASD compared with the general population, notably a significantly higher prevalence and younger age of diagnosis of various chronic conditions. These findings emphasise the need for targeted interventions for ASD adults, with a focus on preventive measures.

# Abstract: Evidence-based practice in primary care: A cross-sectional study of family physicians in Türkiye

**Authors:** Ezgi Agadayi, Duygu Ayhan Başer, Türkü Yağmur Nehir, Ekrem Başara

**Affiliations:** Faculty of Medicine, Sivas Cumhuriyet University, Sivas, Türkiye

**Keywords:** Evidence-based practice, primary care physicians, physicians, family

## Abstract

**Background:**

Evidence-based practice (EBP) is a key component of high-quality primary care. However, the integration of research findings and clinical guidelines into everyday medical practice can be challenging, especially in settings with limited resources. Identifying the specific barriers faced by clinicians is crucial to designing strategies that can promote and facilitate EBP adoption.

**Research questions:**

What are the perceived barriers to implementing EBP among family physicians working in primary care settings in Türkiye?

**Method:**

A descriptive cross-sectional study was conducted between March and December 2024 using an online survey targeting family physicians across Türkiye. The questionnaire included three sections: (1) sociodemographic and professional characteristics; (2) a 13-item list of potential barriers to EBP, rated on a 3-point Likert scale (not affecting, partially, strongly affecting); and (3) the 15-item Evidence-Based Practice Attitude Scale (EBPAS), with items rated from 0 to 4.

**Results:**

A total of 245 family physicians participated. The most commonly reported barriers were limited access to full-text articles (59.6%), lack of time to read current literature (40.8%) and lack of reimbursement for guideline-based care (37.1%). The mean total EBPAS score was 35.0 ± 7.4, indicating moderately positive attitudes. Subscale scores were highest in Divergence (12.0), followed by Appeal (10.4), Openness (8.3) and Requirements (4.4). EBPAS scores showed no significant differences by gender, region, title or patient load. However, age correlated negatively with total score (ρ = −0.24, *p* < 0.001). Physicians who ‘always’ used guidelines had the highest average score (43.0). Open-ended responses frequently mentioned use of Turkish Endocrinology Association guidelines and National Public Health protocols on vaccination and maternal–child care.

**Conclusions:**

While attitudes towards EBP are generally positive, structural and practical barriers persist. System-level interventions are needed to improve access to evidence, encourage guideline use and support younger clinicians in maintaining evidence-informed care.

# Abstract: Learning from the field: A qualitative SWOT analysis of the COVID-19 home vaccination programme in Greece

**Authors:** Dimitra Iosifina Papageorgiou, Despoina Symintiridou, Stavroula Begou, Stella Ploukou, Martha Andreou, Elias Theodoropoulos, Ioannis Vitoris, Marilena Gialama, Panagiotis Stachteas, Ilias Pagkozidis, Chrisovalantis Kakanis, Magda Gavana, Smyrnakis Emmanouil

**Affiliations:** Laboratory of Primary Health Care, General Practice and Health Services Research – Medical School, Aristotle University ofThessaloniki, Thessaloniki, Greece

**Keywords:** Primary care, COVID-19 home vaccination, SWOT framework, Greece

## Abstract

**Background:**

During the COVID-19 pandemic, the COVID-19 home vaccination programme was introduced to reach the frail population unable to access the vaccination points. In Greece, primary care professionals (PCPs) played a pivotal role in delivering this service. This kind of service did not exist prior to the COVID-19 pandemic.

**Research questions:**

What is the experience of PCPs from their participation in COVID-19 home vaccination programme?

**Method:**

A qualitative study was conducted involving semi-structured interviews with PCPs (general practitioners, registered nurses and health visitors) from diverse geographic regions in Greece in the spring of 2022. Participants were purposively sampled based on their profession, the working setting (urban/rural/island) and the sector (public/private). Interviews were audio-recorded, transcribed verbatim and analysed thematically using the SWOT framework to identify internal and external factors shaping their experiences

**Results:**

We analysed 23 interviews. Key strengths included a stimulating experience for the PCPs, increasing collaboration between them and empowering patients and caregivers. Opportunities emerged in terms of enhancing primary care outreach and the interest in the community for the development of home-based primary care programmes for the homebound population. Weaknesses involved logistical challenges, burnout, overworked staff, and limited resources. Finally, threats are identified in the lack of coordination between institutions and social distrust regarding the pandemic and the safety of vaccines.

**Conclusions:**

The participation in the programme highlights how such initiatives, while developed in response to a health crisis, have the potential to be used as a means of strengthening the role of primary care in the community. Addressing barriers emerging either from the healthcare system or from the community is essential to implement similar efforts during future health crises. The SWOT analysis proved to be particularly useful, facilitating the drawing of conclusions and the targeted formulation of proposals for future interventions in home health care.

# Abstract: Point-of-care ultrasonography in Turkish primary care: A qualitative exploration of practice and experience

**Authors:** Öznur Kübra Odabaş, Duygu Ayhan Başer, Adem Özkara

**Affiliations:** Ankara Public Health Directorate, Altındağ, Türkiye

**Keywords:** Family physicians, ultrasonography, Turkey, qualitative study, primary care, point-of-care ultrasound (POCUS)

## Abstract

**Background:**

Ultrasonography (USG) use by family physicians (FPs) is growing worldwide but remains limited in Turkey. There is little research on how FPs incorporate USG into their practice without formal training or institutional support. Understanding their experiences can help guide policy, training and technology integration in primary care. This study explores the motivations, training, practical use and challenges of Turkish FPs who use USG in daily clinical work.

**Research questions:**

What are the experiences, motivations and challenges of FPs using ultrasonography in Turkey?

**Method:**

This qualitative study used a phenomenological approach and COREQ guidelines. Ten FPs from various regions of Turkey were selected through purposive and snowball sampling. Inclusion criteria included at least 6 months of USG use in primary care and having received some form of training. Semi-structured interviews were conducted via video conferencing and transcribed verbatim. Data were analysed thematically using MAXQDA 2020. Themes were developed from the data, revised and adjusted throughout the coding process. Thirteen main themes were identified and supported with participant quotations.

**Results:**

Physicians were motivated by clinical needs, personal interest and gaps in diagnostic access. Education sources varied, with most attending short courses or gaining informal training through clinical experience. Applications included abdominal pain evaluation, pregnancy screening and chronic disease management. Physicians used basic or second-hand equipment. They found USG beneficial for patient care and decision-making. However, some noted that it could increase workload and time pressure, especially in busy primary care settings. Despite challenges, many believed that using USG in family medicine is inevitable in today’s technology-driven healthcare environment.

**Conclusions:**

Ultrasonography is seen as a valuable tool by Turkish FPs, especially in underserved areas. Their experiences show strong motivation but limited support. With better training, policy support and equipment, USG can become a routine part of primary care, improving efficiency, physician confidence and patient satisfaction.

# Abstract: Evaluation of the relationship between frailty, geriatric depression and family functionality in individuals over the age of 65

**Authors:** Cemal Kaygisiz, Yasemin Cayir

**Affiliations:** Faculty of Medicine, Ataturk University, Erzurum, Türkiye

**Keywords:** Elderly, family APGAR scale, frailty, geriatric depression, primary care, ageing

## Abstract

**Background:**

As the population in Türkiye and worldwide continues to age, frailty and depression have emerged as significant public health concerns in older adults. Family functionality plays a key role in maintaining well-being during ageing. From a primary care perspective, understanding the interplay between these factors can inform more holistic and person-centred care strategies.

**Research questions:**

What is the prevalence of frailty and geriatric depression in individuals aged 65 and older? Is there an association between family functionality, frailty and geriatric depression?

**Method:**

This cross-sectional study was conducted in a university-affiliated Family Health Centre in Erzurum, Türkiye, between May and December 2024. A total of 203 individuals aged 65 years and older were surveyed. Data were collected using the Family APGAR Scale, the FRAIL Scale and the Geriatric Depression Scale-Short Form. Descriptive and inferential statistics were used, including *t*-tests, ANOVA and correlation analysis (SPSS v27).

**Results:**

Participants had a mean age of 70.98 ± 4.82 years. Among them, 72.4% had high family functionality, 50.7% were pre-frail, and 41.4% showed signs of depression. There was a significant negative correlation between family functionality and both frailty (*r* = −0.25, *p* < 0.01) and depression (*r* = −0.42, *p* < 0.001), while a moderate positive correlation existed between frailty and depression (*r* = 0.37, *p* < 0.001).

**Conclusions:**

High family functionality is associated with lower levels of frailty and geriatric depression. Strengthening family support mechanisms may improve mental and physical health outcomes in older adults.

# Abstract: Empowering primary care with AI: Leumit advanced predictive health analytics (LAPHA) model improves colorectal cancer detection in a national healthcare setting

**Authors:** Ariel Yehuda Israel, Avivit Golan Cohen, Shlomo Vinker

**Affiliations:** Family Meidicine, Leumit Health Services, Ashdod, Israel

**Keywords:** AI, colorectal cancer, early detection, empowerment of family doctors

## Abstract

**Background:**

Colorectal cancer (CRC) is a leading cause of cancer-related deaths worldwide. Despite the proven benefits of screening, participation remains low, especially among asymptomatic people. New tools are needed to help identify at-risk individuals proactively. Artificial intelligence (AI) provides promising opportunities for personalised screening strategies in primary care.

**Research questions:**

Can an AI-driven model using electronic health records (EHRs) enhance early detection of CRC by prioritising patients for colonoscopy referrals within a primary care-based national health system?

**Method:**

We developed the LAPHA model using de-identified EHR data from Leumit Health Services in Israel. The model analyzes demographic data, diagnoses and longitudinal laboratory test trajectories to estimate CRC risk within 2.5 years. High-risk individuals are proactively referred to colonoscopy through a nurse-led navigation centre. We evaluated CRC detection and polyp yield among individuals undergoing colonoscopy, comparing these rates to the age-matched national incidence.

**Results:**

By July 2025, 685 patients were identified as high risk and contacted. Of these, 322 underwent colonoscopy, revealing 25 cases of CRC (7.8%). In comparison, the CRC incidence in the general age-matched population was 0.08%, resulting in an odds ratio of 108 (95% CI: 40–366). Among the 137 colonoscopy reports retrieved, polyps were found in 76% of cases, and polypectomy was performed in 44% of these cases. These are promising preliminary results from an ongoing implementation.

**Conclusions:**

The LAPHA model significantly boosted the detection rate of CRC and the removal of polyps in a real-world primary care setting. These findings support the incorporation of AI tools into family medicine workflows, providing clinicians with data-driven decision support for preventive care. The approach is scalable, appears resource-efficient and aligns with the evolving role of primary care in population health management.

# Abstract: United by borders, divided by care: The future we leave behind

**Authors:** Emina Bajrić Čusto, Danijel Gajić, Sabina Ćemalović, Senada Selmanović, Sanda Kreitmayer, Branislav Milić, Samir Bajrić, Darko Gajić

**Affiliations:** Health Center Lukavac, Lukavac, Bosnia and Herzegovina

**Keywords:** Health workforce, primary health care, health inequities

## Abstract

**Background:**

Family medicine forms the cornerstone of primary healthcare in the Federation of Bosnia and Herzegovina (FBiH), delivered through 80 health centres across 10 cantons. Despite uniform institutional presence, notable regional disparities exist in the availability of family physicians and specialists, as well as in physicians’ salaries. A comprehensive understanding of these variations is essential to support equitable access and optimise workforce planning.

**Research questions:**

What are the regional differences in the distribution of family medicine physicians and specialists across FBiH cantons? How do these differences correlate with population size, trends in family medicine specialisation and salary disparities?

**Method:**

This descriptive cross-sectional study utilised 2023 administrative health data from all 10 cantons in FBiH. We analysed the number of family physicians, specialists and physicians in training for family medicine, calculating physician-to-population ratios to identify geographic disparities. Regional salary differences were assessed using official payroll data. Age distribution of specialists was also examined to assess workforce demographic structure.

**Results:**

The study revealed substantial regional variation in family medicine workforce density. Urban cantons demonstrated higher ratios of specialists per 100,000 inhabitants, ranging from 5.18 to 33.68 per 100,000 across regions. The total number of family medicine specialists in FBiH is 417, with 157 physicians currently in residency training. The age profile is skewed towards older cohorts, with over half of specialists aged 55–64 and relatively few under 35, highlighting upcoming workforce challenges. Significant salary differences exist, with physicians earning up to 30% more in some cantons, potentially affecting workforce distribution and retention.

**Conclusions:**

Marked regional disparities in family medicine workforce distribution and salaries highlight the need for targeted interventions. These findings underscore the need for strategic workforce planning and enhanced support for specialist training in underserved cantons. This study provides a robust evidence base to inform policy and ensure equitable, sustainable delivery of primary care across the Federation.

# Abstract: Validation of an innovative communication tool for improving compliance in rehabilitation medicine

**Authors:** Pavlo Kolesnyk, Dmytro Hryhorenko

**Affiliations:** Medical Faculty #2, Family medicine and outpatient care department, Uzhgorod National University, Uzhgorod, Ukraine

**Keywords:** Training, family medicine, clinical communication, physical therapy, rehabilitation medicine, patient compliance, empathy, validation, survey, questionnaire

## Abstract

**Background:**

Effective communication in healthcare enhances patient motivation, engagement and compliance. In physical and rehabilitation medicine, structured and empathetic interactions improve outcomes. However, Ukraine lacks easy-to-use mnemonic tools tailored to rehabilitation-specific communication. To address this gap, we developed ‘(KPD)^2^ + ENZiM’, a model combining instructional strategies with elements of patient-centred care. It includes: K–C: Command & Comment; P–P: Plain Phrases & Paraphrase; D–D: Demonstrate & Direct Touch; ENZiM: Empathy, Non-judgement, Zeal (Engagement), Mimics. The model was created within the research project ‘The correlation of compliance and quality of life in patients with post-traumatic pain syndrome and communication skills of medical staff’, supported by the ShowUp4Health grant.

**Research questions:**

How to evaluate the face and content validity of the ‘(KPD)^2^+ENZiM’ communication model in rehabilitation care.

**Method:**

The tool was presented as a mnemonic combining six core interaction strategies and four empathy-based elements. Twenty-six healthcare professionals (clinical communication trainers, PM&R physicians, therapists, nurses and family doctors) rated each element using a 5-point Likert scale. Item Impact Scores (IIS) were calculated. Additionally, 10 experts with over 5 years of experience assessed item relevance, purpose alignment and clarity, allowing calculation of the content validity ratio (CVR) via Lawshe’s method.

**Results:**

The results demonstrated high scores for each component in both the IIS and the CVR, content validity index (CVI). Specifically, IIS scores ranged from 4.77 to 4.92, and CVR values ranged from 0.90 to 1.00, CVI values 0.99, indicating a high level of expert agreement regarding the importance and clarity of the model’s elements. The model received high expert ratings: this reflects strong clarity, relevance and perceived value across all components.

**Conclusions:**

The ‘(KPD)^2^+ENZiM’ demonstrates strong validity and practical relevance in rehabilitation settings. It offers a structured, memorable and empathetic approach to improving communication, enhancing trust and promoting patient adherence in clinical practice.

# Abstract: Factors influencing HPV vaccination behaviour among Bulgarian GPs based on a Pro-VC-Be survey

**Authors:** Gergana Apostolova, Teodora Dimcheva, Zornitsa Ambareva, Radost Assenova

**Affiliations:** Department of Urology and General Practice, Faculty of Medicine, Medical University – Plovdiv, Plovdiv, Bulgaria

**Keywords:** HPV vaccination, general practitioners, vaccine confidence, behavioural determinants, Pro-VC-Be survey

## Abstract

**Background:**

Physician recommendation is a major determinant of HPV vaccine uptake. In Bulgaria, where vaccine hesitancy persists, limited evidence exists on the behavioural and psychosocial factors shaping general practitioners’ (GPs) vaccination practices. The validated Bulgarian version of the Pro-VC-Be questionnaire allows systematic assessment of these determinants.

**Research questions:**

What are the key behavioural drivers influencing HPV vaccination recommendations among Bulgarian GPs, using the Pro-VC-Be tool?

**Method:**

A national cross-sectional online survey was conducted between February and April 2025 among 364 GPs from all regions of Bulgaria. The Pro-VC-Be questionnaire assessed constructs including vaccine confidence, complacency, perceived constraints, collective responsibility and commitment. A Statistical analyses included descriptive statistics, exploratory factor analysis, *t*-tests, ANOVA and multivariate regression to examine associations between Pro-VC-Be scores, vaccination behaviour and demographic characteristics. Data were processed using SPSS v.21.

**Results:**

More than 80% of GPs reported proactive vaccination behaviour and strong awareness of vaccination benefits. Moderate-to-high vaccine confidence and trust in public health authorities. Higher scores in commitment and collective responsibility were significantly associated with routine HPV vaccine recommendation (*p* < 0.01), in contrast perceived constraints such as time pressure, organisational barriers and complacency were negatively associated with vaccination behaviour. Positive vaccination attitudes were also linked to higher self-vaccination rates and willingness to receive future COVID-19 vaccination.

**Conclusions:**

Behavioural determinants play an important role in HPV vaccine advocacy among Bulgarian GPs. Reducing practical barriers and responsibility may strengthen vaccination promotion in primary care.
